# Treatment of glaucoma among elderly patients in Sofia

**DOI:** 10.22336/rjo.2020.62

**Published:** 2020

**Authors:** Milcheva Evelina Marinova, Krasimirova Emilia Naseva, Todorov Yani Zdravkov, Stoimenov Dimitar Dabov

**Affiliations:** * Medical University of Sofia, Bulgaria; ** Department of Ophthalmology, Alexandrovska University Hospital, Sofia, Bulgaria; *** Medical University of Sofia, Faculty of Public Health, Sofia, Bulgaria

**Keywords:** glaucoma, elderly, therapy, fixed combination, medication

## Abstract

**Objective:** To analyze therapy of glaucoma among the elderly population, 65 years old and above, in Sofia city, the capital of Bulgaria.

**Methods:** Two prophylactic ophthalmic campaigns were realized in the summer of 2016 and 2017, in Sofia city. All patients passed thorough ophthalmic examinations and filled pen-and-paper questionnaires. The results and files were collected and statistically analyzed to estimate current antiglaucoma therapy among the elderly citizens of Sofia city.

**Results:** The predominant number of persons who were diagnosed with glaucoma, was treated with conservative monotherapy. Prostaglandin analogues have been prescribed to more than half of the patients in both studies. Fixed combinations have been used in 86% of the cases who needed combined therapy with two substances and in 100% of those with three medications.

**Conclusions:** The therapeutic scheme used for treating glaucoma among elderly citizens of Sofia city is similar and corresponding to practice worldwide. Conservative monotherapy is used in mean 45%. When combined therapy is necessary, fixed combinations are predominantly prescribed with a target to minimize adverse events and to ensure better patients’ compliance. A tendency of usage of preservative-free formulas to benefit patients’ eye health has been noticed.

**Abbreviations:** POSE = prophylactic ophthalmic screening among elderly people, GSW = glaucoma screening week, EGS = European Glaucoma Society, PEX= pseudoexfoliative syndrome, PGAs = Prostaglandin analogues, CAIs = Carbonic Anhydrase Inhibitors

## Introduction

Glaucoma is an irreversible progressive optic neuropathy, for which the major proven treatment is to lower the intraocular pressure (IOP) [**1**-**3**]. It is a generally asymptomatic disease whose prevalence increases with age and reaches 10% over 75 years [**4**]. Predisposing factors are elevated IOP, age, sex, pseudoexfoliations, myopia, diabetes, and family history of glaucoma [**5**]. Six groups of local and systemic medications are used to lower IOP and prevent visual function [**6**]. Topical antiglaucoma drops are predominantly applied [**7**]. Among them, prostaglandin analogues (PGAs) are presenting the highest IOP reduction, followed by β-blockers, and they are licensed for first and second-line use [**3**,**8**]. For the reason that patients’ concordance and drop instillation techniques are of great importance for the result of the treatment, conservative therapy with single medication is preferred by the ophthalmologists if it could ensure a good control of the disease [**9**]. When monotherapy is insufficiently effective, the alternatives include changing medication, the addition of an extra drug, performing laser or surgery [**10**,**11**]. Fixed combination drugs ensure better patient adherence with a simple dosing regimen and less exposure to preservatives when multiple medications have to be applied [**5**,**12**]. Laser treatment is used in 10% of the cases to ensure pressure control without local medication, in addition to the current therapy, and in the cases with closed-angle glaucoma. Surgery has to be performed when medications and laser treatment are insufficiently effective or in advanced glaucoma at diagnosis [**13**]. 

## Methods

The study population included the elderly citizens of Sofia city (65 and above), who were enrolled for analysis after entering 2 screening programmes organized by the Ophthalmology Department of Alexandrovska University Hospital in Sofia, Bulgaria.

An announcement for prophylactic ophthalmic examinations of elderly Sofia citizens, over the age of 65, was transmitted through mass media in two consequent years (2016 and 2017), for 3 weeks during summer. The study was named POSE = Prophylactic ophthalmic screening among the elderly.

A month later, another prophylactic campaign, Glaucoma screening week (GSW) was organized. 

All persons passed a thorough ophthalmic examination, including visual acuity test, slit-lamp biomicroscopy, intraocular pressure measurement, fundoscopy using stereoscopic slit-lamp biomicroscopy with 90D fundus lens. The attendants filled paper-and-pen questionnaires about their current complaints and therapy, usage of correction glasses and refraction, diagnosed systemic and ocular diseases, family, and medical history. These data revealed tendencies in anti-glaucoma treatment, used by the ophthalmologists in Sofia.

Statistical methods

A descriptive statistic was applied. Qualitative variables were presented as numbers and percentages, numerical variables were represented as the median and interquartile range (both 25 and 75 percentile). The relation between numerical variables was checked by Spearman’s rho correlation coefficient. 

## Results

Prophylactic ophthalmic screening among elderly people (POSE) study

**Fig. 1:**
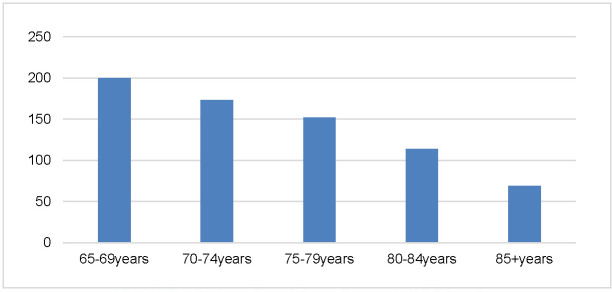
Distribution of participants by age in POSE study

800 individuals from the general population responded to the invitation. 708 of them, (1415 eyes) were persons aged 65 years and older but not evenly distributed by age. The biggest number of participants (n=200), 28%, were aged 65 to 69. Only 69 of the attendants, (9.7%) were over 85 years (**Fig. 1**).

**Fig. 2:**
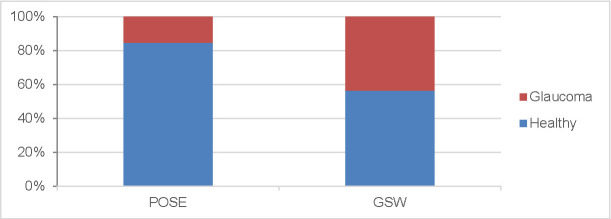
Distribution of pseudoexfoliations (PEX) among patients who attended in POSE study

A fifth of the attendants (n=142, 20.3%) have had at least one of these characteristics: elevated IOP over 21 mmHg, narrow anterior ocular chamber, Cup/ disk ratio over 0,5 or inequality of C/ D ratios of both eyes over 0.2, a glaucomatous look of the optic nerve head and pseudoexfoliative syndrome. A thorough ophthalmic examination was strongly recommended to all of them. Of all 708 persons, 23 have had a narrow anterior chamber angle and 66 have had a pseudoexfoliative syndrome, with prevalence increasing with age (**Fig. 2**).

A thorough ophthalmic examination was offered to 28 persons who were strongly suspected to have glaucoma because of the presence of more than two clinical signs of the disease. 16% of the participants (99 patients) with a median age of 75 years (IQR 70-80), have been previously diagnosed with glaucoma. Less than half of them, 43% (n=43), were males. 7% of them (n=7) have had secondary glaucoma (neovascular, traumatic, inflammatory); 14% (n=14) have had pseudoexfoliative glaucoma; 4% (n=4) have had closed-angle glaucoma;10% (n=10) have had glaucoma surgery procedures; laser treatment has been previously performed in 9 persons (9%); information about the treatment of 9 of glaucoma patients (9%) was not received. 

Glaucoma screening week (GSW)

A month later, another prophylactic campaign, named Glaucoma screening week (GSW), was organized and transmitted through mass media. Of all the patients who entered the screening, 78 were over 65 years old. 34 of them have had glaucoma. 

**Fig. 3:**
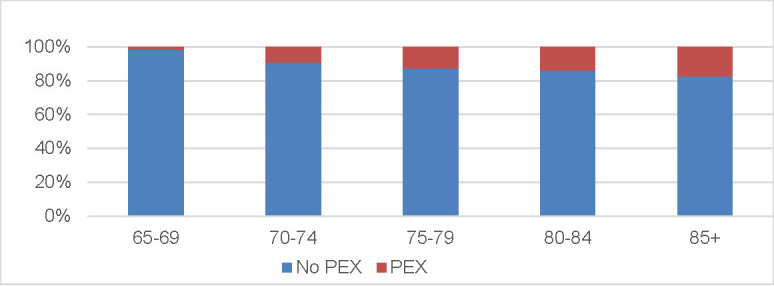
Prevalence of persons without glaucoma and patients previously diagnosed with glaucoma in POSE and GSW studies

The prevalence of glaucoma among the elderly participants in GSW was found to be 44% but in POSE it was 14% (**Fig. 3**).

45 attendants in POSE and GSW have been additionally observed in Alexandrovska University Hospital. Some of them entered the clinic for a routine follow up of previously diagnosed glaucoma, reevaluation of target IOP, and current therapy and control of the rate of progression.

Another group has had poor control of the disease with signs of progression or an elevated IOP and the rest were patients suspected to have glaucoma. All the hospitalized patients have passed a thorough ophthalmic examination for glaucoma including visual acuity with correction, measurements of central corneal thickness, and visual field using standard automated perimetry, diurnal IOP curve (using Goldmann applanation tonometry), gonioscopy, slit-lamp biomicroscopy and funduscopy with 90D fundus lens, and optical coherence tomography. In cases with progression or IOP higher than the target, therapy was optimized by changing the medication, the addition of an extra drug, laser treatment, or surgical procedures. 15 of the hospitalized patients were suspected to have glaucoma. After the previously discussed examinations, 10 of them were diagnosed with glaucoma and started treatment. Monotherapy with a prostaglandin analogue (PGA) was prescribed to 4 patients (40%), 3 received β-blockers, and one patient received CAI. Combined therapy was prescribed to two persons (20%) because of the insufficient effect of monotherapy. Both patients received β-blocker plus PGA, one in a fixed combination, and the other separated, in 2 containers.

Among patients from POSE, previously diagnosed with glaucoma, a predominant number, of almost 68% were using Prostaglandin analogues (PGAs), whereas in the GSW group, they were 54%. 55% were taking β-blockers in mono- or combined therapy compared to 85% in GSW. 19% did not receive therapy at the moment, because they have already gone through different kinds of surgical procedures, including laser treatment (**Table 1**).

**Table 1:** Distribution of the usage of different groups of antiglaucoma medications in mono- or combined therapies in both studies

Group of medication	Percent, % in POSE	Percent, % in GSW
PGAs	68%	54%
β-Blockers	55%	85%
CAIs	24%	39%
α-2 agonists	9%	8%
No conservative therapy	19%	10%

When discussing the number of drugs used to control IOP it becomes visible that monotherapy was predominantly used for treating 43% of the glaucoma patients in the POSE study. The comparison between the current anti-glaucoma therapies of the participants, examined in POSE, and those from Glaucoma screening week (GSW) emphasized some points to consider. The usage of monotherapy in both studies was almost equal, 46% in GSW, compared to 43% in POSE. There was a highlighting difference in the usage of combined therapy between the studies. The percentage of patients who were using two medications from GSW was near to double compared to the persons with an identical regimen from POSE. The higher number of used substances correlated to lower prevalence (**Table 2**).

**Table 2:** Analysis of the number of medications used to treat glaucoma

Number of medications	Percent, % in POSE	Percent, % in GSW
Monotherapy	43%	46%
Two medications	22%	39%
Three medications	10%	8%
Four medications	5%	8%

A thorough look at the number of containers of eye drops, irrespective of the number of active substances, used from the patients with glaucoma, collected in both screening programmes, showed almost equal data. Mean 64% were using a single-container therapy and 14% were using two bottles of eye drops (**Table 3**). 

**Table 3:** Number of containers of eye drops used by the patients

Number of containers	Percent, % in POSE	Percent, % in GSW
Single container	63%	65%
Two containers	15%	12%
Three containers	3%	0%

**Table 4** emphasizes on the use of fixed combinations when more than one medication is necessary for treating glaucoma. Anti-glaucoma therapy in one container was used by mean 78%. It could be concluded that at least one fixed combination is used when the number of active substances exceeds the number of containers of eye drops. Fixed combinations were prescribed to 86% of the patients who needed two medications therapy and in 100% of the cases with triple therapy. In cases that needed three or more medications to control the IOP, at least one fixed combination was prescribed (**Table 4**).

**Table 4:** Correlation between the number of active substances and the number of containers of eye drops

Number of medications *Number of containers			
Number of medications	Number of containers		
	1	2	3
Monotherapy	100%	0%	0%
Two medications	86%	14%	0%
Three medications	0%	100%	0%
Four medications	0%	40%	60%
Total	78%	19%	4%

The analysis of the groups of used medication revealed that β-blockers monotherapy was applied in thirty percent, but in 70% they were included in combined therapy. Monotherapy with PGAs was registered in 44% but in 56%, PGAs were used in combined therapy. CAIs were prescribed in only 4% as monotherapy whilst in 96% they were included in polydrug therapy. α-2 agonists were found in 56% in combined therapies of 4 drugs, thus it could be concluded that Bulgarian ophthalmologists have generally prescribed them as the last option for conservative treatment (**Table 5**). 

**Table 5:** Usage of different groups of antiglaucoma drugs in mono and combined therapies

Group of medication	Number of drugs used			
	1	2	3	4
No β-blockers	31%	3%	0%	0%
β-blockers	30%	41%	20%	9%
No PGAs	21%	8%	0%	0%
PGAs	44%	30%	18%	8%
No CAIs	36%	14%	1%	0%
CAIs	4%	33%	42%	21%
No α-2 agonists	32%	17%	7%	0%
α-2 agonists	11%	22%	11%	56%

Lots of patients were switched to therapy with preservative-free formulas either as monotherapy or in a fixed combination of eye drops to diminish adverse events of chronic exposition to preservatives in continuous glaucoma treatment with different classes of drugs.

The relation between the age and the number of containers was checked and a very strong correlation was proved (Spearman’s rho=0.891, p=0.014).

The analysis of anti-glaucoma therapy by age revealed that PGAs were the predominantly used medications, followed by β-blockers. The biggest prevalence of conservative local treatment was in the group 75-79 years old, in which PGAs were used in almost 49% and β-blockers in 43%. In all the other age groups, the prevalence of the same medication classes was lower. Monotherapy was prescribed mostly in the group 65-69 years old and its usage was diminishing with age, while the need for combined therapies was increasing with increasing age. The highest level of usage of combined therapy was found in the group 75-79 years old.

## Discussion

The paradigm for the early detection and treatment of the diseases, especially asymptomatic, is a basis of general medicine that has a great impact on the ophthalmology, especially when discussing glaucoma. The disease is more often found among the elderly population, risk increasing with age (by 26% per decade), which is caused by many different mechanisms of influence. Meanwhile, this group of the society has had many difficulties in receiving access to ophthalmic consultation like lots of comorbidities, problems with movement, low incomes, diminishing mental capacity, etc. These findings make the elderly people a target group for preventive programmes and investigations to estimate morbidity and analyze treatment. Screening programmes are cheap and easy to organize but they have a great impact not only on the life of every person who was diagnosed and received a chance to keep his vision, but also on society, by diminishing the number of blind or visually impaired persons [**14**]. All these facts forced our team to organize screening programmes to estimate the prevalence of different ophthalmic diseases and their treatment, to ensure easy access to medical help for this group of the society and to analyze tendencies in morbidity and treatment.

The POSE study could be considered as representing a sample of “the third age” of Sofia, because of the great number of attendants of 65 years old and older who felt healthy related to their ocular condition. On the other hand, GSW was announced as screening for glaucoma. When comparing files of people over 65 years old from both studies, a highlighting difference in the distribution of individuals, previously diagnosed with glaucoma was found. The relative percentage of glaucoma patients was significantly higher in GSW than in POSE study. These patients have had more severe forms that needed aggressive treatment and usage of a greater number of drugs to maintain the target IOP and glaucoma control. Although the group of elderly patients in GSW was small and not statistically significant, we supposed that the announcement of health campaigns was targeting different groups of society. When examinations were organized as screening for a concrete disease, a higher prevalence of this state should have been expected to be found. 

According to the data presented above, conservative treatment was used in most cases, with a predominance of monotherapy with PGAs, followed by beta-blockers. Preservative-free formulations were preferred, especially in cases with ocular surface disease or allergy. When combined therapy was necessary, fixed combination eye drops were chosen because of lower exposure to preservatives and to ensure better patient compliance and adherence. The majority of the patients were using a single-container therapy. Laser treatment had been applied in cases with allergy, intolerance, poor compliance, or with angle-closure glaucoma, pupillary block, or when target IOP could not be reached with conservative therapy [**7**,**9**,**10**,**15**-**17**].

## Conclusion

The great number of persons suspected to have had glaucoma, 5% of all attendants in POSE study, and 10 newly diagnosed patients (1,5%), who started treatment, emphasized the need for regular prophylactic examinations for the elderly population as a whole, not only for the target groups.

The therapeutic scheme used for treating glaucoma, ocular hypertension, and glaucoma, suspected in ambulant and hospital practice in Bulgaria, strongly corresponded to practice worldwide and the guidelines published by many professional ophthalmology organizations and institutions. The rising number of persons who use preservative-free eye drops and the tendency that more patients are switched to those formulations, improve their compliance and the state of the anterior ocular surface.

**Acknowledgments**

None.

**Sources of Funding**

None.

**Disclosures**

None.
